# Comprehensive transcriptome profiling of *Salvia miltiorrhiza* for discovery of genes associated with the biosynthesis of tanshinones and phenolic acids

**DOI:** 10.1038/s41598-017-10215-2

**Published:** 2017-09-05

**Authors:** Wei Zhou, Qiang Huang, Xiao Wu, Zewen Zhou, Mingquan Ding, Min Shi, Fenfen Huang, Shen Li, Yao Wang, Guoyin Kai

**Affiliations:** 1 College of pharmacy, Zhejiang Chinese Medical University, Hangzhou, Zhejiang, 310053 China; 2 Laboratory of Plant Biotechnology, College of Life and Environment Sciences, Shanghai Normal University, Shanghai, 200234 China; 3 The Key Laboratory for Quality Improvement of Agricultural Products of Zhejiang Province, School of Agriculture and Food Science, Zhejiang A&F University, Linan, Hangzhou, Zhejiang, 311300 China

## Abstract

Tanshinones and phenolic acids are crucial bioactive compounds biosynthesized in *Salvia miltiorrhiza*. Methyl jasmonate (MeJA) is an effective elicitor to enhance the production of phenolic acids and tanshinones simultaneously, while yeast extract (YE) is used as a biotic elicitor that only induce tanshinones accumulation. However, little was known about the different molecular mechanism. To identify the downstream and regulatory genes involved in tanshinone and phenolic acid biosynthesis, we conducted comprehensive transcriptome profiling of *S. miltiorrhiza* hairy roots treated with either MeJA or YE. Total 55588 unigenes were assembled from about 1.72 billion clean reads, of which 42458 unigenes (76.4%) were successfully annotated. The expression patterns of 19 selected genes in the significantly upregulated unigenes were verified by quantitative real-time PCR. The candidate downstream genes and other cytochrome P450s involved in the late steps of tanshinone and phenolic acid biosynthesis pathways were screened from the RNA-seq dataset based on co-expression pattern analysis with specific biosynthetic genes. Additionally, 375 transcription factors were identified to exhibit a significant up-regulated expression pattern in response to induction. This study can provide us a valuable gene resource for elucidating the molecular mechanism of tanshinones and phenolic acids biosynthesis in hairy roots of *S. miltiorrhiza*.

## Introduction

*Salvia miltiorrhiza* Bunge (Dan shen in Chinese) is a traditional Chinese herb with significant medicinal and economic values. It has been used widely to treat many cardiovascular diseases such as menstrual disorder, blood circulation disturbance, inflammation and angina pector^1^. The active ingredients of *S. miltiorrhiza* can be divided into two groups: one group is water-soluble phenolic acids, such as rosmarinic acids (RAs), salvianolic acids and lithospermic acid, functions as antibacterial, anti-oxidative and antiviral reagents^2^; the other group is diterpenoid tanshinone, including tanshinone I, tanshinone IIA, tanshinone IIB, dihydrotanshnone I and cryptotanshinone, exhibits various pharmacological activities including antioxidant, antitumor and anti-inflammatory properties. The active constituents obtained from cultivated *S. miltiorrhiza* is low and cannot meet the rapidly increasing market need^3, 4^. Genetic manipulation of active ingredients biosynthesis pathway in plants or hairy roots provide us a promising strategy^5^, which rely on the detail understanding of the biosynthesis pathway and regulation mechanism.

The unique chemical constituents of *S. miltiorrhiza*, diterpenoids tanshinones are derived predominantly from the plastidic methylerythritol phosphate (MEP) pathway or partly through the cytoplasmic mevalonate (MVA), with possible cross-talk between the common precursors of isopentenyl diphosphate (IPP) and dimethylallyl diphosphate (DMAPP)^4–6^. The phenolic acids in *S. miltiorrhiza* are synthesized via the tyrosine-derived and phenylpropanoid pathways^7^.

Until now, some key enzyme genes in the upstream of biosynthesis pathways of bioactive compounds in *S. miltiorrhiza* have been cloned and characterized by conventional cloning methods with slow speed^8–12^. To overcome the limitations of conventional methods, next generation (NG) sequencing methods such as Illumina, provide an easy way for quick analysis of one organism’s transcriptome with numerous genes. Some studies have attempted to elucidate the entire biosynthesis processes of bioactive compounds in *S. miltiorrhiza* by analyzing its transcriptome and have cloned several transcription factors (TFs) associated with the biosynthesis and regulation of these bioactive compounds^13–18^. It is reported that some cytochrome P450 proteins, decarboxylase, dehydrogenase and reductase probably participate in the catalytic reaction steps in the downstream biosynthesis of tanshinones through analyzing the structure traits of downstream compounds. But the exact information about the downstream enzymes catalyzing the several steps from ferruginol to tanshinones in tanshinone biosynthesis pathway and from rosmarinic acid to lithospermic acid in phenolic acids biosynthesis pathway is still unknown (Supplementary Fig. S1).

Methyl jasmonate (MeJA) and yeast extract (YE) are proposed to be effective elicitors to increase the accumulation of tanshinones and phenolic acids. Most of the genes involved in these pathways are also induced by MeJA or YE^19^. As we found that MeJA was an effective elicitor to enhance the production of phenolic acids and tanshinones simultaneously, while YE was an elicitor only for tanshinones accumulation. Little is known about the different molecular mechanism lay behind. To identify the downstream and regulatory genes involved in tanshinone and phenolic acid biosynthesis, we conducted comprehensive transcriptome profiling of *S. miltiorrhiza* using hair roots treated with MeJA or YE by RNA-seq strategy, respectively. Some important candidate downstream biosynthetic genes and regulatory genes were predicted. It would be an interesting work for functionally identifying the downstream biosynthetic genes in the biosynthesis of tanshinones and phenolic acids and elucidating the molecular mechanism of MeJA-mediated and YE-mediated biosynthesis of tanshinone and phenolic acid.

## Results and Discussion

### Transcriptome sequencing, de novo assembly and sequence clustering

The sequencing of five *S. miltiorrhiza* hairy root cDNA libraries generated 37,866,226 raw reads from the non-elicitor treated control, 42,574,704 from MeJA-1h (Sample was induced by MeJA for one hour), 42,514,138 from MeJA-6h, 49,822,250 from YE-1h and 54,383,514 from YE-12h. Approximately 25% of raw reads were removed post filtering of adapter sequences, low quality reads and short reads. Further, 27364554 valid reads in control, 32801610 in MeJA-1h, 32161100 in MeJA-6h, 38973026 in YE-1h and 41483248 in YE-12h were assembled de novo using trinity^20^, which resulted in total 55588 unigenes. The statistical summary of data is outlined in Table 1. The average unigene length was 1292.04 bp, GC content was 49% and N50 was 1772 bp.

**Table 1:** Summary of *S. miltiorrhiza* transcriptome.

	Control	MeJA-1h	MeJA-6h	YE-1h	YE-12h	All Unigene
Raw Reads	37866226	42574704	42514138	49822250	54383514	—
Clean Reads	27364554	32801610	32161100	38973026	41483248	—
Number of contigs	46354	48181	44857	51660	46708	55588
Number of characters	40707368	40872184	39108985	45165688	44225189	71821817
Maximum contig length(bp)	11988	12449	12002	10940	12280	12449
Minimum contig length(bp)	201	201	201	201	201	300
Median contig length(bp)	589	558	580	588	646	1022
Average contig length(bp)	878.18	848.31	871.86	874.29	946.84	1292.04
Contig N50 length(bp)	1340	1304	1338	1348	1461	1772
GC% content after filtering (%)	49	49	49	49	49	49
Reads mapping to All-Unigene (%)	97.61	97.68	97.95	97.44	97.81	—

### Unique sequence annotation

Unigenes were searched against non-redundant protein database (NR) in NCBI and the Swiss-Prot database and 42458 unigenes had at least one match to known protein sequences. After consolidation, 76.4% unigenes had been functionally annotated. *S. miltiorrhiza* belongs to *Lamiaceae* family, the whole genome of this family has not been assembled nicely so far^20^. This may be one of the reasons for relatively low homology results. Although EST database of *S. miltiorrhiza* was developed earlier but only reported very few unique transcripts^21, 22^. The sequences with unknown homology may represent genes involved in metabolic processes which are unique to this plant and whose intermediates and enzymes have not been identified so far. Moreover, transcriptome studies of other plant species have also reported functional annotation of about half of unigenes. For example, only 49.88% of the unigene could be annotated in transcriptomes of *Cymbidium sinence* ^23^. Further, significant match could be assigned to only 55% of the unigenes in bamboo transcriptome^24^ and only 52.89% of the unigenes from *Amaranthus tricolor* showed significant similarity to known genes^25^.

### Differential gene expression analysis and functional classification

In *S. miltiorrhiza* hairy root cultures, methyl jasmonate (MeJA) was an effective elicitor to enhance the production of phenolic acids and tanshinones, while yeast extract (YE) was only effective for tanshinones biosynthesis. In order to identify the different molecular mechanism of MeJA-mediated and YE-mediated biosynthesis of tanshinone and phenolic acid, we studied differential gene expression (DGE) between MeJA-induced and YE-induced *S. miltiorrhiza* hairy root.

**Figure 1:**
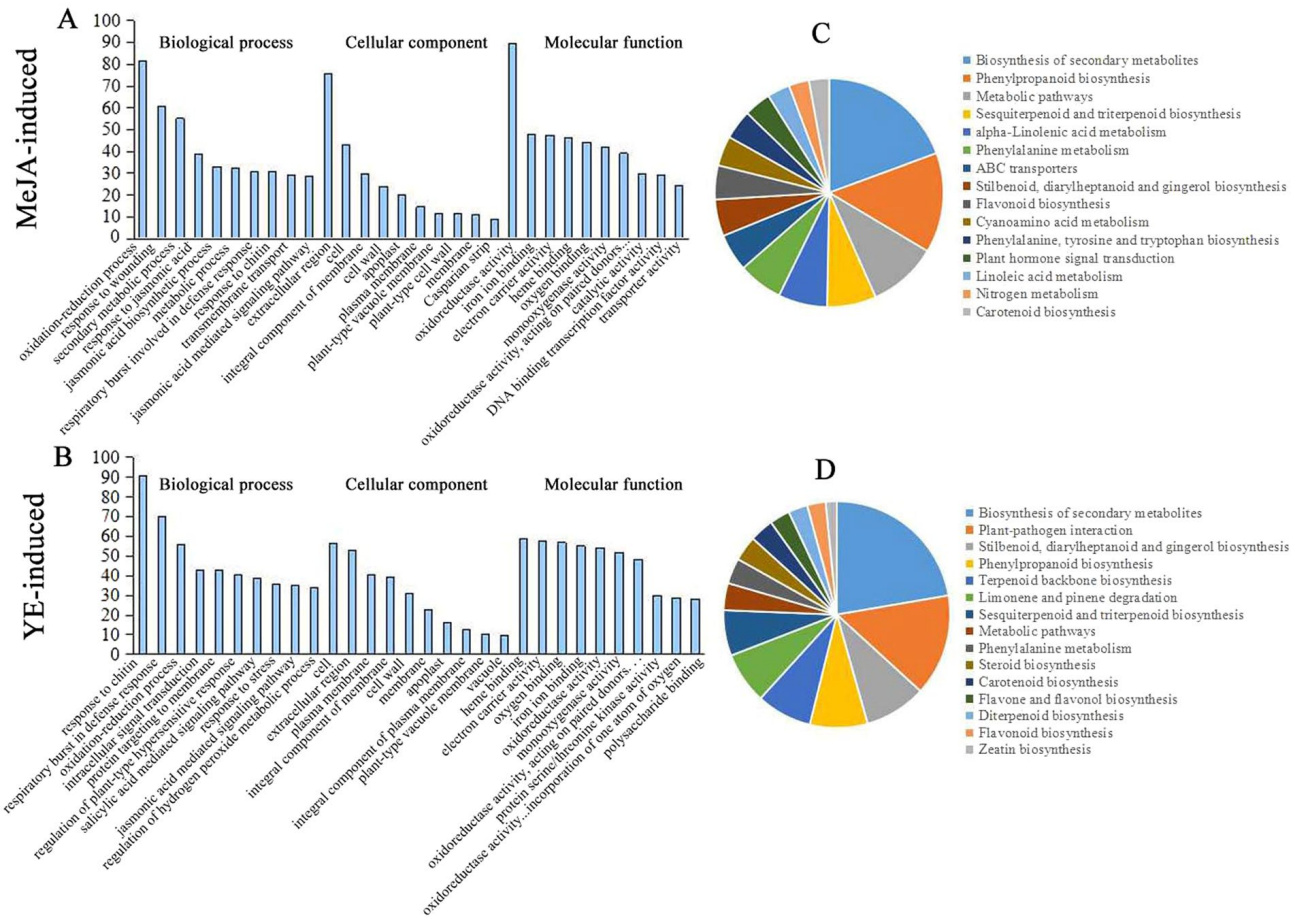
GO and KEGG analysis of unigenes by MeJA and YE. (**A**,**B** bar chart shows GO terms associated with unigenes up-regulated by MeJA and YE separately. (**C**,**D**) pie chart representing KEGG classes of MeJA and YE- induced differently expression genes).

We calculated the normalized expression values Reads Per Kilobase per Million mapped reads (RPKM) of each unigene, and those with > 2-fold change and a false discovery rate (FDR) < 0.01 were considered as differentially expressed genes^26^. Total of 5767 unigenes in MeJA-induced transcriptome and 4482 unigenes in YE-induced transcriptome were significantly upregulated (Supplementary Table S1). Gene ontology (GO) enrichment analysis was carried out to identify the major functional categories represented by differentially expressed genes. We observed that in MeJA-induced upregulated genes, the top five classes contributed to ‘biological process’ were oxidation-reduction process, response to wounding, secondary metabolic process, response to jasmonic acid and jasmonic acid biosynthetic process (Fig. 1A). As to the genes upregulated in YE-induced transcriptome, response to chitin, respiratory burst involved in defense response, oxidation-reduction process, intracellular signal transduction and protein targeting to membrane were the top five categories (Fig. 1B). In the molecular function category, the top five classes represented by MeJA- and YE-induced upregulated genes were all oxidoreductase activity, iron ion binding, electron carrier activity, heme binding and oxygen binding (Fig. 1A,B). Kyoto enclyclopedia of genes and genomes (KEGG) analyses of differentially expressed genes showed that genes involved in biosynthesis of secondary metabolites, phenylpropanoid biosynthesis, metabolic pathways, sesquiterpenoid and triterpenoid biosynthesis, and alpha-linolenic acid metabolism were predominantly enriched in MeJA-induced transcriptomes (Fig. 1C), while in YE-induced transcriptome, biosynthesis of secondary metabolites, plant-pathogen interaction, stilbenoid, diarylheptanoid and gingerol biosynthesis, phenylpropanoid biosynthesis, and terpenoid backbone biosynthesis represented the major classes (Fig. 1D). YE can be treated as a biotic elicitor and MeJA is a hormone elicitor. YE can promote the biosynthesis of tanshinones, while MeJA is effective for accumulation of both tanshinones and phenolic acids^5, 6^. The number of DEGs related to the biosynthesis of secondary metabolites, phenylpropanoid biosynthesis, metabolic pathways, sesquiterpenoid and triterpenoid biosynthesis mediated by plant hormone signal transduction in MeJA-induced transcriptome is significantly more than in YE (Supplementary Table S2), while in case of YE-induced transcriptome, genes related to plant-pathogen interaction and terpenoid backbone are much more than in MeJA-induced (Supplementary Table S3). In fact, several studies confirmed that YE had a more significant influence on the genes involved in sesquiterpenoid and triterpenoid biosynthesis while MeJA had a remarkable effect on the genes involved in phenylpropanoid, phenylalanine, tyrosine and tryptophan metabolic pathways, which is consistent with the effect of YE and MeJA on the biosynthesis of tanshinone or/and phenolic acid^3, 19^. The different enrichment of YE and MeJA induced transcriptome implied that these genes responding to tanshinone and phenolic acid biosynthesis mainly exists in biosynthesis of secondary metabolites, phenylpropanoid, metabolic pathways, sesquiterpenoid and triterpenoid, plant hormone signal transduction and plant-pathogen interaction classes. These annotation processes for DGEs provide a valuable resource for identifying specific biosynthetic genes and related genes in signal transduction pathways that respond to MeJA and YE in *S.miltiorrhiza* separately, especially for providing useful information to examine the fine mechanism of plant secondary metabolites in *S. miltiorrhiza*.

### Content of tanshinones and phenolic acids by HPLC

**Figure 2:**
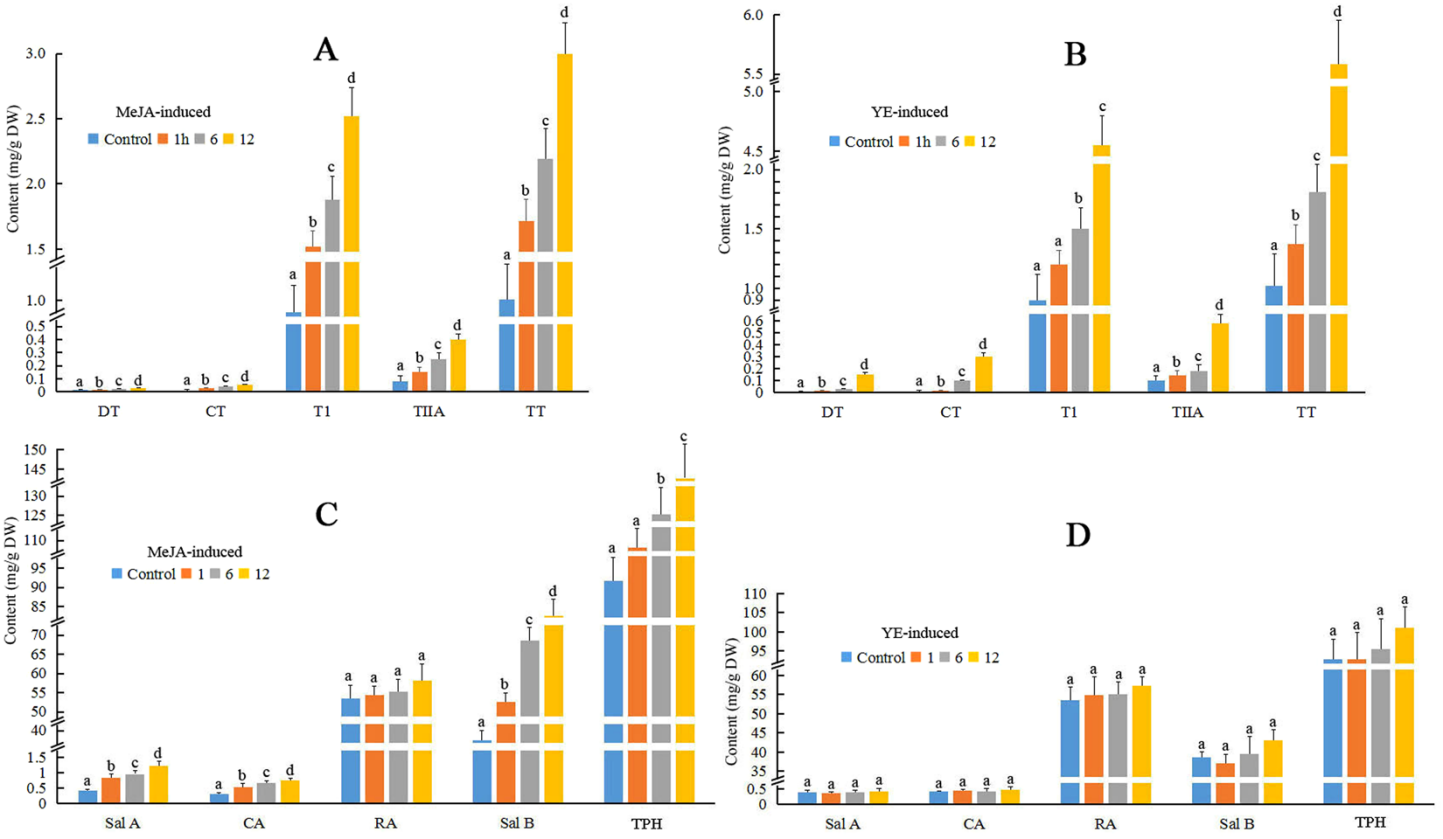
Content of tanshinones and phenolic acids assayed by HPLC. ((**A**,**C**) hairy root lines induced by MeJA; (**B**,**D**), hairy root lines induced by YE. Error bars represent standard error of the mean. The lower case letters above the column show the content of tanshinones and phenolic acids have a variance that are significantly different, based upon *T*-tests (*p* < 0.05); DT, dihydrotanshinone; CT, cryptotanshinone; T1, tanshinone I; TIIA, tanshinone IIA; TT, total of tanshinones; SalA, salvianolic acid A; CA, caffeic acid; RA, rosmarinic acid; SalB, salvianolic acid B; TPH, total of phenolic acids).

The total content of tanshinones (TT) and phenolic acids (TPH) in *S. miltiorrhiza* hairy roots were detected by HPLC. Tanshinones (including DT, CT, T1 and T2A) got a significant increase in hairy roots induced by MeJA and YE separately than the control. For YE-induced hairy roots, a maximum 4.47 folds increase of total tanshinones (TT) was observed, and a 1.94 folds increase were found in MeJA-induced hairy roots. MeJA, but not by YE, could induce the content of phenolic acids (TPH comprising of Sal A, Sal B and CA). For the hairy roots induced by MeJA, the total content of phenolic acid (TPH) got a maximum 0.56 fold increase compared with the control (Fig. 2).

### MeJA and YE responsive genes related to tanshinones and phenolic acids biosynthesis

Previous studies found that MeJA and YE can induce the expression of genes involved in the biosynthesis of tanshinones and phenolic acids and lead to the accumulation of these compounds in *S. miltiorrhiza* ^3, 19^. To elucidate the whole molecular mechanism of MeJA and YE-mediated tanshinones and phenolic acids biosynthesis, the expression of genes involved in tanshinones and phenolic acids biosynthesis were investigated. We observed 66 unigenes that encoded 26 enzymes involved in the biosynthetic pathways of tanshinones. In addition, we identified 37 unigenes that encoded 17 enzymes involved in the biosynthetic pathways of phenolic acid. These genes encode the enzymes involved in the MEP/MVA pathway and the downstream pathway for the biosynthesis of tanshinone, which include *DXS*, *DXR*, *CMK*, *MCS*, *HDS* and *IDS* in MEP pathway, *AACT*, *HMGR*, *HMGS*, *MK, PMK* and *MDC* in MVA pathway and *IPPI*, *GGPPS*, *GPPS*, *FPPS*, *KSL*, *CYP76AH1* and *CYP76AH3* in the downstream pathway for tanshinone biosynthesis (Supplementary Table S4). Almost all the genes in the biosynthesis pathway for tanshinone identified in our transcriptome dataset were confirmed by previous studies^9–12^. Compared to MeJA, YE had a more significant effect on genes involved in biosynthesis of tanshinone, which got 50 unigenes (encoded 19 enzymes) having RPKM values >2 folds change more than the control while 33 unigenes (encoded 18 enzymes) got the RPKM values change 2-fold more than the control induced by MeJA (Supplementary Table S4 and Supplementary Fig. S2). The expression level of *KSL* response to MeJA is consistent with the result obtained by Ma *et al*.^12^, but was not coincided with Luo *et al*.^16^, which may be caused by the different treated plant materials.

Unlike this, the expression patterns of genes involved in phenolic acid biosynthesis pathways were diverse. *PAL*, *C4H*, *4CL*, *TAT*, *HPPR*, *RAS* and *CYP98A14* were all induced by MeJA (with the maximum 13.6 folds RPKM values changes compared to the control). Nevertheless, YE didn’t show any clear effect on these genes. It was also consistent with change of active ingredients contents after treatment by these two elicitors, in which the content of tanshinones in hairy root could be induced by these two elicitors, but to phenolic acid, the contents could only be induced by MeJA, not by YE (Fig. 2).

### Experimental validation of differential expressed genes by qRT-PCR

**Figure 3:**
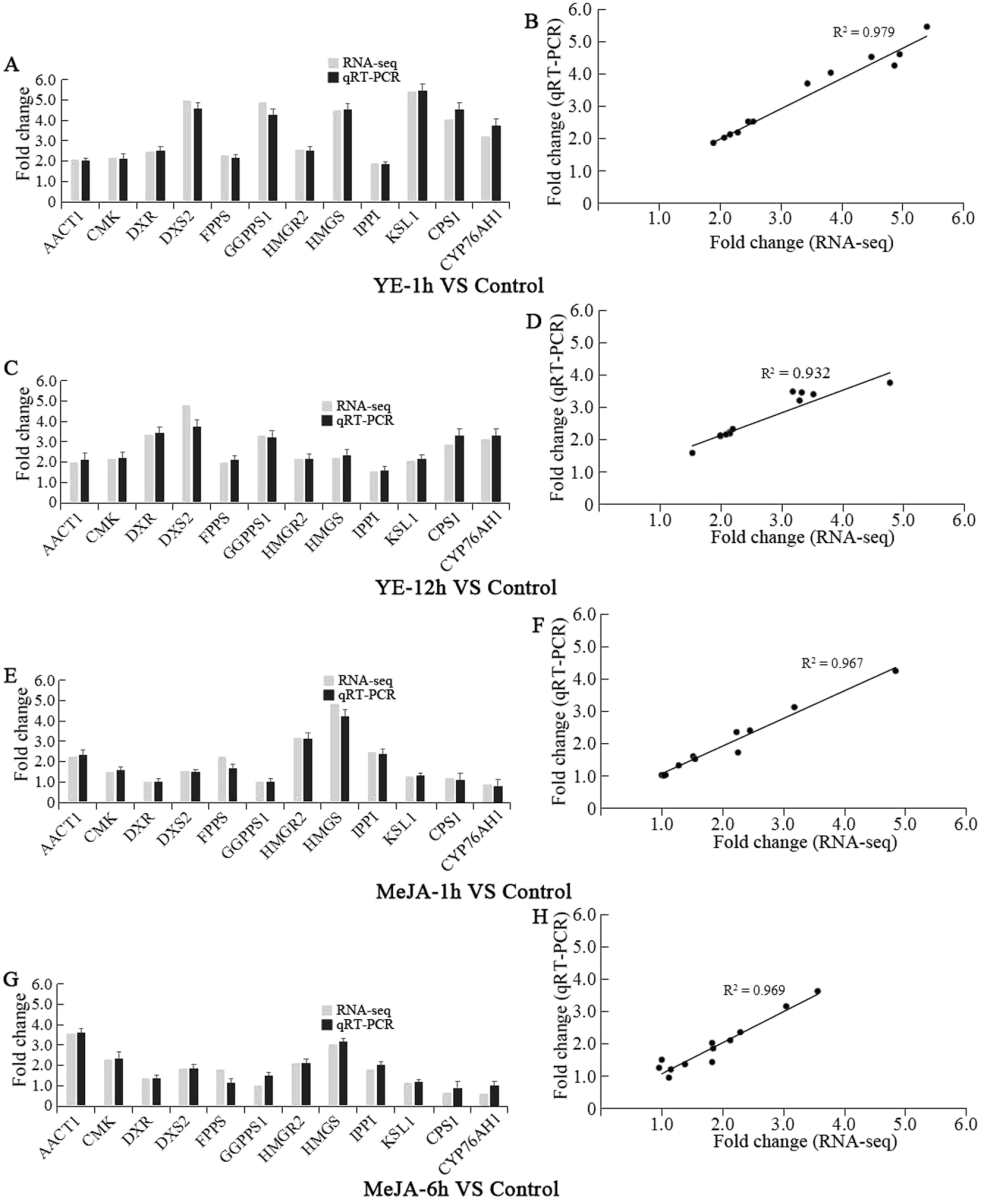
qRT-PCR validation of differently expressed genes in RNA-seq dataset. (**A**,**C**,**E** and **G** Expression of 12 genes was validated using qRT-PCR to compare with their expression in RNA-seq. Error bars indicate standard error of the mean. (**B**,**D**,**F** and **H)** Correlation of gene expression results obtained from qRT-PCR and RNA-seq).

qRT-PCR was introduced to validate the differential gene expression obtained by RNA-seq. Twelve genes involved in tanshinone biosynthesis including *AACT1*, *CMK*, *DXR*, *DXS2*, *FPPS*, *GGPPS1*, *HMGR2*, *HMGS*, *IPPI*, *KSL1*, *CPS1* and *CYP76AH1*, were selected for qRT-PCR. The expression pattern of the these twelve genes as obtained by qRT-PCR was in line with those obtained by RNA-seq data. Statistical analysis a nice correlation between the two data was observed, with the correlation coefficient ranging from 0.932 to 0.979 (Fig. 3). Furthmore, severn genes involved in phenolic acids biosynthesis including *PAL1*, *TAT1*, *4CL1*, *HPPR1*, *C4H1*, *RAS1* and *CYP98A14* were also selected for qRT-PCR detection. The result showed a nice coherence between the results of qRT-PCR and the dataset obtained by RNA-seq (Supplementary Fig. S3). It implied that the dataset obtained by RNA-seq is reliable for exploring the target downstream genes and regulatory genes involved in the biosynthesis of tanshinones and phenolic acids.

### MeJA-responsive and YE-responsive P450 genes in *S. miltiorrhiza*

**Figure 4:**
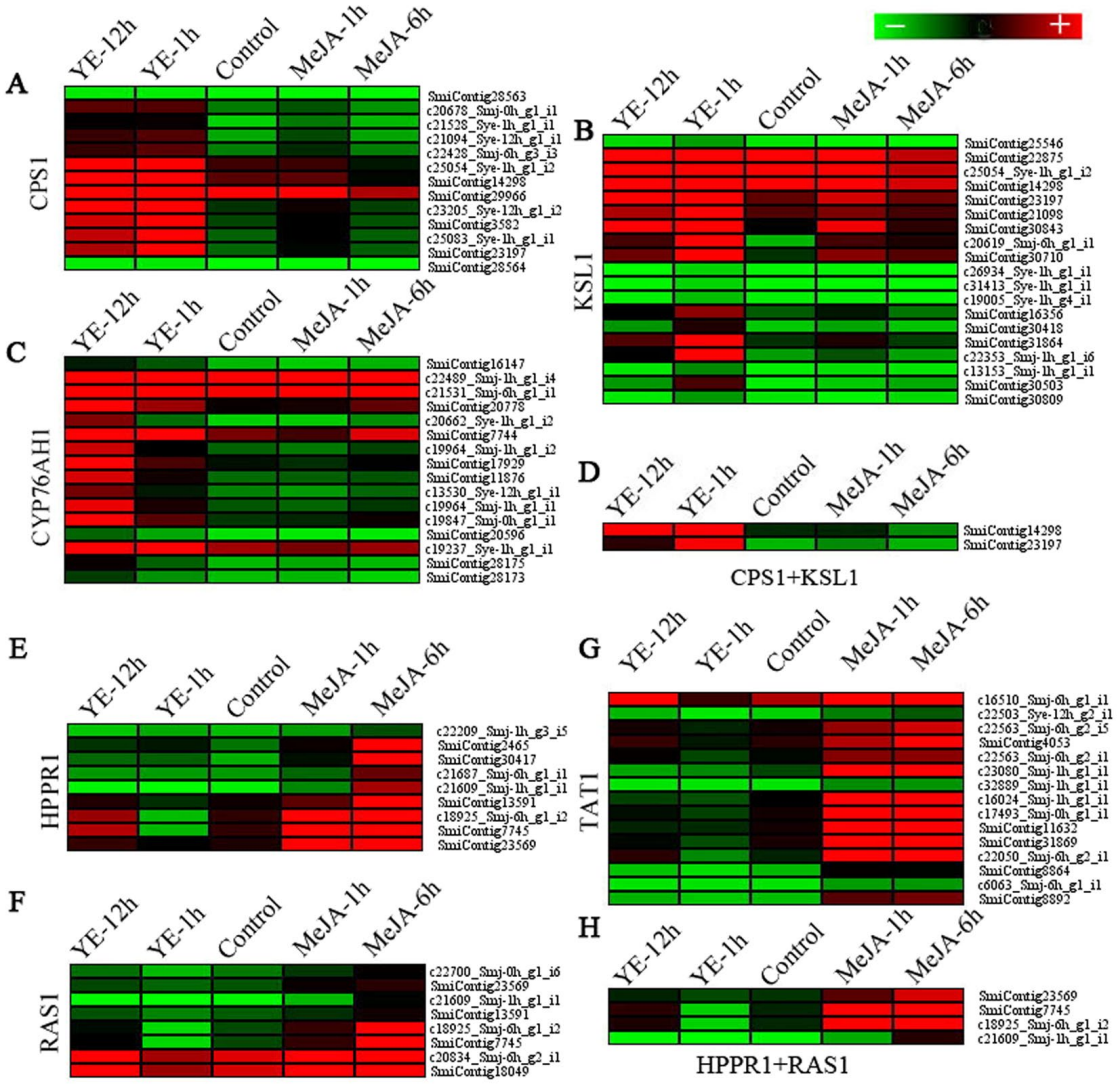
Co-expression analysis of P450 genes with downstream biosynthetic genes involved in the biosynthesis of tanshinones and phenolic acids. (**A**–**H** Co-expression with CPS1, KSL1, CYP76AH1, KSL1 + CPS1, HPPR1, RAS1, TAT1, HPPR1 + RAS1, separately).

Cytochrome P450s being one of the biggest gene super families of plants, participate in various biochemical pathways especially in secondary metabolic pathways, including phenolic compounds, flavonoids, isoprenoids and alkaloids^27, 28^. Co-expression analyses revealed that some P450s are coordinately expressed with key biosynthetic genes to constitute biological pathways. The combination of high-throughput transcriptome sequencing and co-regulation analysis allows for the formulation of highly precise hypotheses on the function of uncharacterized biosynthetic genes^26, 27^. Previous reports elucidated that the expression of key genes such as *CPS1*, *KSL1* and *CYP76AH1* positively correlated with the tanshinone accumulation in hairy roots of *S.miltiorrhiza* under the treatment of MeJA and YE respectively^3, 6, 15^. In this study, total of 45 candidate P450 unigenes in MeJA and 27 unigenes in YE-induced transcriptome were selected from the up-regulation P450 members based on co-expressed pattern analysis with the downstream biosynthetic genes involved in the biosynthesis of tanshinones, including *CPS1*, *KSL1* and *CYP76AH1*, among of which 19 P450 unigenes showed a co-expression pattern with *KSL1*, 13 P450 unigenes with *CPS1*, 16 P450 unigenes with *CYP76AH1* and only two P450 unigenes with both *KSL1* and *CPS1* (Supplementary Table S5 and Fig. 4A–D).

In the pathway of phenolic acid biosynthesis, total of 26 P450 unigenes exhibiting a co-expression pattern with genes in the metabolic pathway of phenolic acids (including *TAT1*, *HPPR1* and *RAS1*), were selected from up-regulation P450 members (RPKM values > 2 folds change between elictor treatment and control), with 15 unigenes co-expressed with *TAT1*, 9 unigenes co-expressed with *HPPR1*, 7 unigenes co-expressed with *RAS1*, 4 unigenes co-expressed both with *HPPR1* and *RAS1* (Supplementary Table S6 and Fig. 4E–H). A cytochrome P450-dependent monooxygenase (CYP98A14), which can introduce 3-hydroxyl group in the late pathway to form RAs, was identified in *S. miltiorrhiza* ^29^. A P450, named CYP76AH1 that catalyzes a unique four-electron oxidation cascade on miltiradiene to produce ferruginol, an important intermediate compound for tanshinone biosynthesis, was also recently characterized^30^. The expression levels of these above two P450s were significantly induced by MeJA, and the studies indicated that P450s have key functions in tanshinone and phenolic acid biosynthesis^29, 30^. Thus, the identification of these P450s contributes to the elucidation of the downstream biosynthetic pathways for tanshinone and phenolic acid biosynthesis.

**Figure 5:**
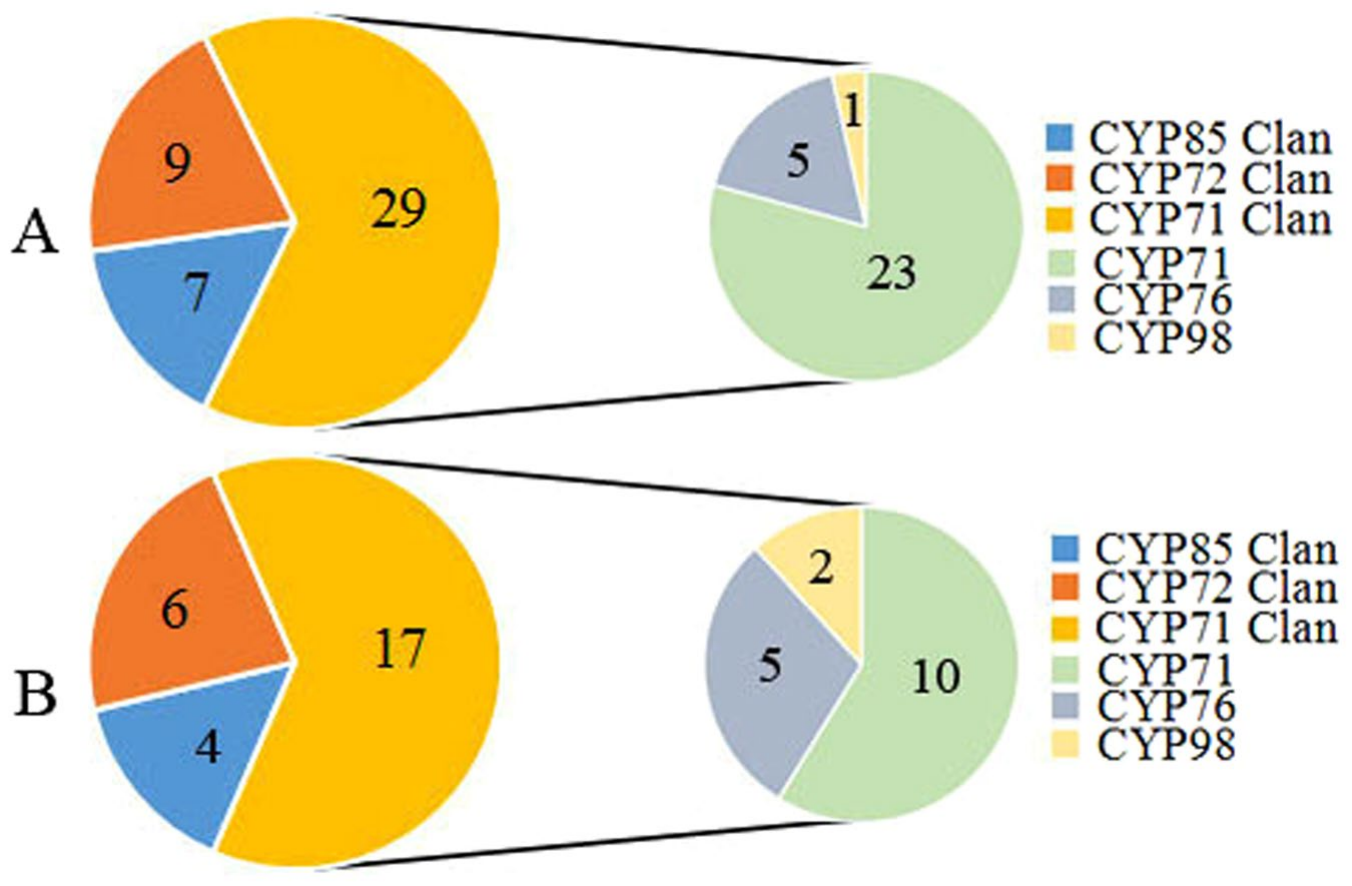
Candidate cytochrome P450 genes for tanshinones and phenolic acids biosynthesis.(**A**, Distribution of the 45 up-regulated CYPs in clans, as well as the family distribution of the 29 members from the CYP71 clan; (**B**) Distribution of the 27 up-regulated CYPs in clans, as well as the family distribution of the 17 members from the CYP71 clan).

CYP71 clan P450s was thought to contain the most P450 members and was known to be involved in plant secondary metabolism^27^. In order to gain insight into the phylogenetic relationship between various P450s, a neighbour-joining phylogenetic tree was constructed with the deduced protein sequences of these selected P450s (45 P450 unigenes in MeJA and 27 P450 unigenes in YE-induced transcriptome) and P450s from other plant species. For P450 genes in tanshinones biosynthesis pathway, 29 unigenes in CYP71 clan (23 unigenes in CYP71 family, 5 unigenes in CYP76 family and 1 unigenes in CYP98 family), 9 unigenes in CYP72 clan and 7 unigenes in CYP85 clan were identified (Fig. 5A and Supplementary Fig. S4). To P450 genes in phenolic acids biosynthesis pathway, 17 unigenes in CYP71 clan (10 unigenes in CYP71 family, 5 unigenes in CYP76 family and 2 unigenes in CYP98 family), 6 unigenes in CYP72 clan and 4 unigenes in CYP85 clan were also identified (Fig. 5B and Supplementary Fig. S5). In rice, a diterpenoid biosynthetic gene cluster was identified on chromosome 2, in which two genes encoding CYP71 clan members of CYP76M7 and CYP71Z7 catalyze the early and later steps in phytocassane biosynthesis, respectively^31, 32^. Thus, the *S. miltiorrhiza* CYP71 clan members can be of particular interests in further elucidation of biosynthetic pathway of tanshinones. Due to distinct catalytic activities of P450 monooxygenases, differentially expressed P450 genes of the CYP85 and CYP72 clans also hold the potential to be enzymes of the pathway.

### Downstream of candidate genes involved in tanshinone and phenolic acid biosynthesis

Due to the hypothetical tanshinones biosynthetic pathway deduced by Yang *et al*.^15^ and Guo *et al*.^17^, biosynthesis of tanshinones involved in the downstream steps of decarboxylation, oxidation and reductions could be catalyzed by decarboxylase, dehydrogenase and reductase, respectively^8, 15, 17, 30^. Unigene dataset was searched with *Zingiber zerumbet* short-chain dehydrogenase (ZSD1, AB480831) and *Artemisia annua* aldehyde Δ11(13) reductase 2 (DBR2, EU704257) to identify candidate dehydrogenases and reductases^33, 34^. 7 unigenes and 6 unigenes showed more than 42% protein sequence identity to ZSD1 and DBR2 respectively (Table 2). All the unigenes were expressed at significantly higher levels in elicitor treatment (MeJA or YE separately) after than before, with the maximum 6.96 folds change of RPKM values (FDR < 0.01), suggesting a high possibility of the dehydrogenase and reductase being involved in the biosynthesis of tanshinones.

**Table 2:** Expression of candidate downstream genes involved in the biosynthesis of tanshinones and phenolic acids.

Gene	Unigene ID	MeJA-1h	MeJA-6h	Control	YE-1h	YE-12h
Reductase	c23543_Smj-1h_g1_i2	321.25	184.58	91.40	75.48	112.25
	c4200_Smj-1h_g1_i1	51.46	28.91	25.91	19.84	26.72
	SmiContig16951	59.49	30.04	24.95	19.78	30.17
	SmiContig32803	397.26	184.30	129.36	143.80	161.71
	SmiContig6622	410.93	122.53	59.05	58.43	46.86
	SmiContig32802	232.74	45.01	47.77	171.43	78.49
Dehydrogenase	c16637_Smj-1h_g1_i2	10.86	3.96	4.90	14.30	4.78
	SmiContig20215	8.04	16.59	6.90	18.64	14.31
	SmiContig29584	11.32	1.89	3.16	9.49	2.58
	SmiContig29586	6.31	2.02	2.59	6.50	1.37
	SmiContig29955	8.66	3.29	3.29	10.15	3.57
	SmiContig26874	6.16	3.72	2.13	3.67	1.82
	SmiContig32840	106.37	86.31	33.41	29.99	47.66
Laccase 1 A	SmiContig4158	82.34	144.07	62.91	22.78	41.17
	c22112_Smj-6h_g1_i1	332.55	816.08	55.05	38.96	52.13
	SmiContig23578	213.25	378.59	42.47	37.30	43.50
	SmiContig4159	299.17	592.16	93.07	44.28	69.83
Laccase 2	SmiContig18382	206.52	266.23	112.76	138.61	181.98
	SmiContig6627	67.93	61.91	33.64	33.82	45.33
	SmiContig8962	19.50	1.19	9.25	11.16	9.32

For the biosynthesis of phenolic acids, it was confirmed that rosmarinci acid was the substrate of salvianolic acid B and the biosynthetic step was thought to be a catalytic reaction of dimerization^29^. Ponzoni *et al*.^35^ reported that laccases derived from *Trametes pubescen*s, could catalyze hydroxystilbene to form a dimerization reaction and produce three types of analogues (A, B and C), among of which A-type metabolite had a structure similar to salvianolic acid B. It implied that laccase might be a candidate enzyme to catalyze rosmarinci acid to form salvianolic acid B. Therefore, unigenes dataset was searched with *T. pubescens* laccase 1 A (Lap1A, AF414808) and laccase 2 (Lap2, AF414807) to identify candidate laccase involved in the biosynthesis of phenolic acids^36^. Total of 4 unigenes being similar to *Lap1A* and 3 unigenes being similar to *Lap2*, were all identified (Table 2).

### MeJA and YE-responsive TFs in *S. miltiorrhiza*

In plants, transcription factors (TFs) of different families regulate a series of life process, including development, evolution and the response to abiotic and biotic stresses. Many TFs have been found to regulate secondary metabolite biosynthesis and accumulation^37^. One of the aims of the present work was to identify TFs which regulate the biosynthesis and accumulation of tanshinone and phenolic acid induced by YE and MeJA in *S. miltiorrhiza* hairy roots. The unigenes encoding TFs in our RNA-seq dataset were investigated. RPKM values of all the TFs in the elicitation treatment and the control were compared, which resulted in total 375 TFs with > 2-fold (FDR < 0.01) expression levels in YE or MeJA elicitation treatment than the control (Supplementary Table S7). Our dataset have more up-regulated TFs than Gao *et al*.^14^ and Yang *et al*.^15^, it might be due to different samples treated by different style. But the fact that the four major TFs showing a up-regulated expression pattern including WRKY, bHLH, AP2-EREBP and NAC is simlar to the previous reports by Gao *et al*.^14^ and Yang *et al*.^15^, for which it implied their important effects in the biosynthesis of tanshinones and phenolic acids mediated by YE and MeJA (Supplementary Fig. S6).

**Figure 6:**
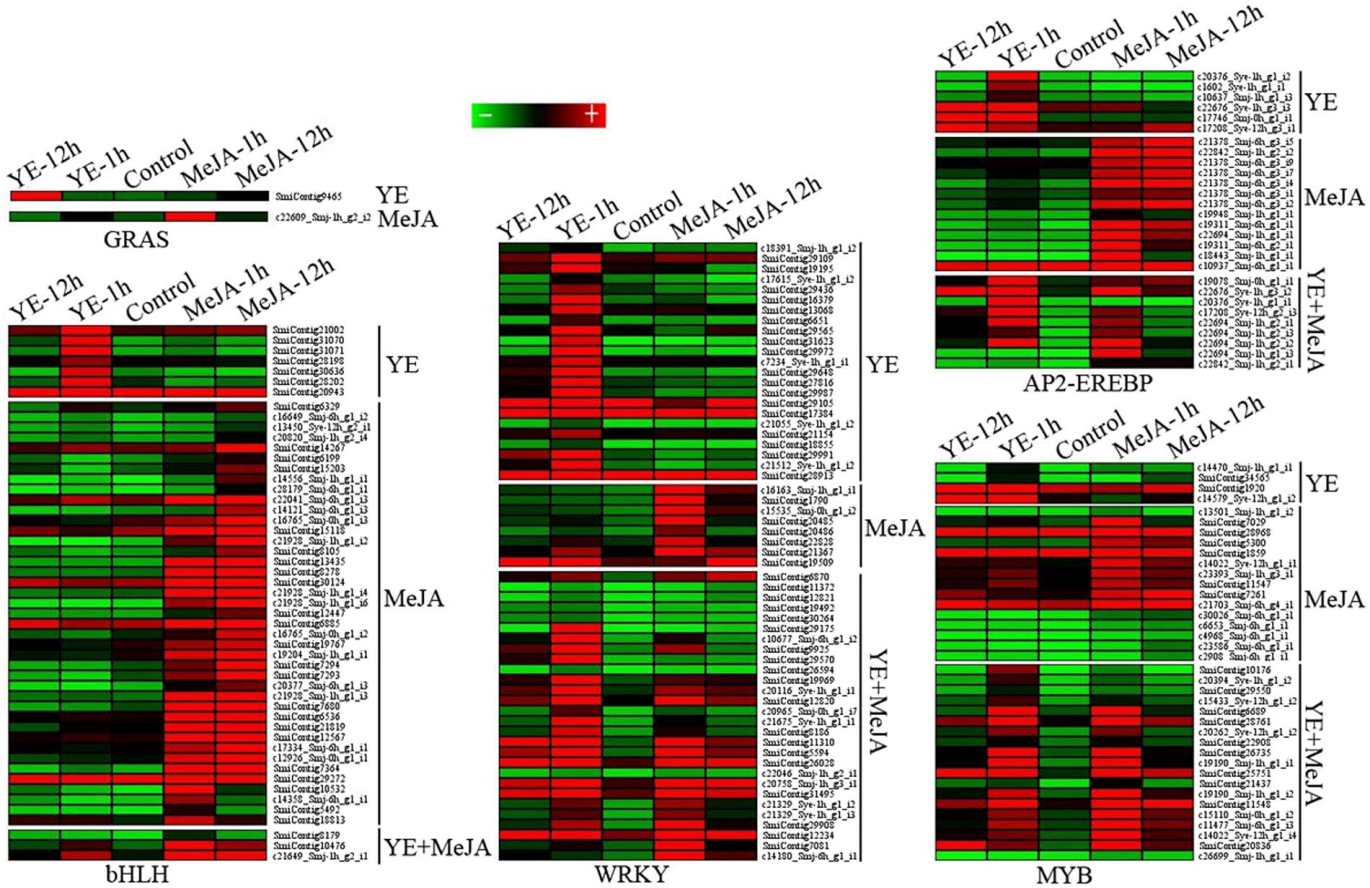
Expression of transcriptional factors (TFs) involved in tanshniones and phenolic acids biosynthesis. The heat graph shows TFs differential expression induced only by YE or MeJA, or by both MeJA and YE.

Previous studies reported that TFs including WRKY, AP2-EREBP, MYB, GRAS and bHLH families showed more important roles in the regulation of plant secondary metabolism^14, 16, 37^. In this study, we found that 59 WRKY, 28 AP2-EREBP, 38 MYB, 2 GRAS and 51 bHLH were up-regulated in YE- and/or MeJA-mediated transcriptomes, among which 23 WRKY, 6 AP2-EREBP, 4 MYB, 1 GRAS and 7 bHLH were only up-regulated by YE, not by MeJA; Reversely, to MeJA-mediated transcriptome, 8 WRKY, 13 AP2-EREBP, 15 MYB, 1 GRAS and 41 bHLH were up-regulated only by MeJA (Fig. 6 and Supplementary Table S7).

Among these up-regulated genes, AP2-EREBP, MYB and bHLH were more abundant in *S. miltiorrhiza* hairy roots treated with MeJA than those treated with YE, while WRKY family was more abundant in YE treated trascriptome which indicated that AP2-EREBP, MYB and bHLH families probably showed a more positive effect in participating the MeJA-mediated signal transduction and secondary metabolite biosynthesis, but WRKY families showed a reverse result in YE-mediated signal transduction and metabolism. The bHLH protein can physically interact with certain MYBs and WD-repeats and acts as an important regulator to modulate anthocyanin accumulation in plant species^38^. Thus TFs responding to MeJA- and YE-mediated elicitation might interact with each other to regulate secondary metabolite biosynthesis and accumulation of tanshinone and phenolic acid. WRKY protein (CjWRKY1) is a transcriptional regulator of benzylisoquinoline alkaloid biosynthesis in *Coptis japonica* ^39^. The AP2-EREBP members have been shown to regulate secondary metabolism pathways in several medicinal plants. In *Catharanthus roseus*, the AP2-EREBP proteins *ORCA2* and *ORCA3* positively regulate the expression of strictosidine synthase (*CrSTR*) and the biosynthesis of terpenoid indole alkaloid^33^. In *Artemisia annua*, two JA-responsive AP2-EREBP proteins, *AaERF1* and *AaERF2*, participate in artemisinin metabolism by binding to the promoter of *AaADS* and *AaCYP71AV1* genes, and finally activate their expression^40^. In our dataset, 13 and 7 AP2-EREBP members were induced by MeJA and YE respectively in which some members were even elevated to dozens of times compared to the control. It implied that AP2-EREBP TFs might have a remarkable effect on secondary metabolite biosynthesis and accumulation in *S. miltiorrhiza* hairy roots.

Despite 375 TFs in *S. miltiorrhiza* hairy roots that be responsive to YE or MeJA elicitation were identified, it is possible that not all these TFs participate the biosynthesis of tanshinones and/or phenolic acids directly and the range of target TFs could by shortened by Weighted Gene Co-expression Network Analysis (WGCNA) strategy further^26, 41, 42^. In fact, some similar research works on TFs have been studied in *Arabidopsis thaliana*, *Catharanthus roseus*, *Solanum tuberosum* and other plants^14, 16, 33, 41, 42^. However, some TFs associated with regulation of the biosynthesis of tanshinones and phenolic acids, which were not up-regulated significantly by YE or MeJA, would be missed out in this study. Therefore it is important that identification of key biosynthetic genes and TFs using various strategies could help us to dissect the molecular mechanism underlying the biosynthesis of tanshinones and/or phenolic acids in the future. In a word, this work provides us a useful gene resource for discovery and functional identification of downstream biosynthetic genes and upstream TFs involved in the biosynthesis of tanshinones and phenolic acids.

## Materials and Methods

### Hairy root culture

Sterile *S. miltiorrhiza* plants were grown and maintained in our laboratory as reported previously^9, 14^. To induce hairy roots, sterile leaf sections were used for infection with the disarmed *A. tumefaciens* strain C58C1 (pRi A4) as reported before by our groups^10^. The hairy root clones were routinely subcultured every 30 days on solid 1/2 MS medium. Rapidly growing hairy roots with normal growth status were used to establish hairy root lines, and the liquid 1/2 MS medium were changed every 20 days. Forty-day-old shake-flask cultured hairy roots were treated with elicitors, and the hairy roots were harvested from the culture medium at selected times after treatment by MeJA (0 h, 1 h and 6 h) or YE (1 h and 12 h) separately^4–6^. All the induced hairy root lines were harvested for RNA isolation.

### Preparation of MeJA and YE elicitors

MeJA was dissolved in ethanol followed by filter-sterilization and added to the culture medium at a final concentration of 0.1 mM. A total of 25 g yeast extract from Bio Basic Inc was dissolved with 125 ml distilled water, and then yeast elicitors were prepared by two rounds of ethanol precipitation as reported before^43^.

### RNA isolation, quantification and qualification

Total RNAs from different hairy root lines were extracted using the *TRIzol* Reagent (Invitrogen) and treated with *DNase* I (Takara) according to manufacturer’s protocols^44^. Each sample was prepared by mixing three replicate samples. RNA were monitored using EtBr-stained 1% agarose gel. The RNA purity and concentration were determined using a Nanodrap spectrophotometer (Thermo).

### cDNA library preparation and sequencing

Reverse transcription was performed with the SMART II ^TM^ cDNA Synthesis Kit (Clontech, USA) following the manufacturer’s instructions. Double strand cDNAs were separated on 2% agarose gel, and those with size over 100 bp were recovered. The RNA-seq and construction of the libraries were performed by Novelbio Biotechnology Corporation (Shanghai, China) and the cDNA library was sequenced using Illumina HiSeq^TM^2000. All reads were deposited in the Short Read Archive (SRA) of the National Center for Biotechnology Information (NCBI) public database under the accession GSE100970.

### Data analysis and assembly

The sequence reads is processed to remove adapter sequences, low quality reads and very short length reads. Paired reads were quality filtered using NGS QC toolkit v 2.3^45^. The cutoff for quality score is > 20 Q30 score with high quality bases > 70% of read length. High quality reads were used for de novo assembly using Trinity software with K-mer of 25. The assembly resulted in contigs and singletons which together form set of unigenes.

### Unique sequence annotation and functional classification

Unigenes were used as query sequences to search against the non-redundant protein (NR) database at NCBI (http://www.ncbi.nlm.nih.gov) and the Swiss-Prot protein database (http://www.ebi.ac.uk/uniprot) with *E*-value cutoff of 1e^−5^. The annotations of the best hits were recorded. Gene Ontology (GO) (http://www.geneontology.org/) was further used to category the function of the unigenes by Blast2GO^46^, and the unigenes were assigned to biological functions on the macro levels of ‘biological process’, ‘cellular component’ and ‘molecular function’. The unigenes were assigned by KEGG Automatic Annotation Server (KAAS) upon the KEGG pathways database (http://www.genome.jp/kegg/)^47^.

### Differential expression and co-expression network analysis

For differentially expressed genes assay, reads per kilobase per million reads (RPKM) values were used to normalize gene expression levels. Statistical comparison of RPKM values between elicitors-induced and non-induced sample was performed using software Bowtie^48^. Statistical test analysis was done using FDR method^49^. The corrected *P*-value (false discovery rate, FDR) of 0.05 was set as the threshold for significant differential expression. A gene expression correlation network between the candidate synthetic enzyme genes in the tanshinones and phenolic acids biosynthesis pathway and candidate P450s or transcriptional factors was constructed with the Weighted Gene Co-expression Network Analysis (WGCNA) method^26^, co-expression correlation was defined based on the following criteria: FDR ≤ 0.001 and *P*-value < 0.01. One color bar represents a range of coefficient values; the symbol ‘-’ denotes the declining expression pattern and the symbol ‘ + ’ denotes the increasing expression pattern. Hierarchical clustering was used to examine the correlation between the elicitors-induced and non-induced samples by the K-means clustering method. Heat map was produced by the software Heatmap 2.0^50^.

### Determination of tanshinones and phenolic acids by high-performance liquid chromatography (HPLC)

Elicitors-induced and non-induced hairy root lines were harvested and dried. Dried hairy roots (200 mg) were pulverized and extracted with 16 mL of methanol/dichloromethane (3:1, v/v), sonicated for 1 h and then kept at room temperature for 24 h. Isolation of tanshinones and phenolic acids for HPLC analysis were performed as reported before^13^. Four tanshinone species comprising of dihydrotanshinone (DT), cryptotanshinone (CT), tanshinone I (T1) and tanshinone IIA (T2A), and four phenolic acids including Salvianolic acid A (Sal A), Salvianolic acid B (Sal B), Caffeic acid (CA) and Rosmarinic acid (RA) were detected and quantified by comparison with authentic standard curves and retention times. Total tanshinone comprising of DT, CT, T1 and T2A, and total phenolic acids comprising of Sal A, Sal B, CA and RA were designated as TT and TPH separately in this paper.

### Validation of RNA-Seq data by quantitative real-time RT-PCR (qRT-PCR)

First strand cDNA was synthesized from 100 ng total RNA using reverse transcriptase (Takara, Japan). For each reaction, 1/10th of the RT reaction mixes was used as the template for PCR. The sequences of primers used in this study are shown in Supplementary Table S8. Real-time PCR was performed using SYBR Pre-mix *Ex* Taq™ II kit (Promega, Beijing) and run on Step One Software v2.0 (ABI Step One, USA). Gene expression were calculated in relation to the reference gene *18srRNA* using 2^–ΔΔCT^ method^51^. Each generated data point represented the average of three independent biological replicates.

### Data availability statement

The authors declare that all the data in this manuscript is available.

### Ethical Standard

The experiment conducted complies with the laws of China.

## Electronic supplementary material

Supplementray Figure S1-S6

Supplementray Table S1

Supplementray Table S2

Supplementray Table S3

Supplementray Table S4

Supplementray Table S5

Supplementray Table S6

Supplementray Table S7

Supplementray Table S8
