# Supplementary material for: Comprehensive transcriptome profiling of Salvia miltiorrhiza for discovery of genes associated with the biosynthesis of tanshinones and phenolic acids
Source: Sci Rep. 2017 Sep 5;7:10554. doi: 10.1038/s41598-017-10215-2 (PMC5585387; doi:10.1038/s41598-017-10215-2)
Supplement: Supplementary file 1 — Supplementray Figure S1-S6 [file 41598_2017_10215_MOESM1_ESM.docx]

**Supplementary Information**

**Comprehensive transcriptome profiling of *Salvia miltiorrhiza* for discovery of** **genes associated with the biosynthesis of tanshinones and phenolic acids**

Wei Zhou^c, a+^, Qiang Huang^b+^, Xiao Wu^b^, Zewen Zhou^b^, Mingquan Ding^c^, Min Shi^b^, Fenfen Huang^b^, Shen Li^c^, Yao Wang^b^, Guoyin Kai^a,b^*

^a^*College of pharmacy, Zhejiang Chinese Medical University, Hangzhou, Zhejiang 310053, China.*

^b^*Laboratory of Plant Biotechnology, College of Life and Environment Sciences, Shanghai Normal University, Shanghai 200234, China;*

*^c^The Key Laboratory for Quality Improvement of Agricultural Products of Zhejiang Province, School of Agriculture and Food Science, Zhejiang A&F University, Linan, Hangzhou, Zhejiang 311300,China.*

***^+^*** *These authors contributed equally to this work*

**Corresponding author: Guoyin Kai, Ph.D, Professor; Address: Laboratory of Plant Biotechnology, College of Life and Environment Sciences, Shanghai Normal University, 100 Guilin Road, Shanghai 200234, P.R.China; Tel/Fax: 86-21-64321291; E-mail:* [*guoyinkai@yahoo.com*](mailto:guoyinkai@yahoo.com)*,* [*gykai@hotmail.com*](mailto:gykai@hotmail.com)


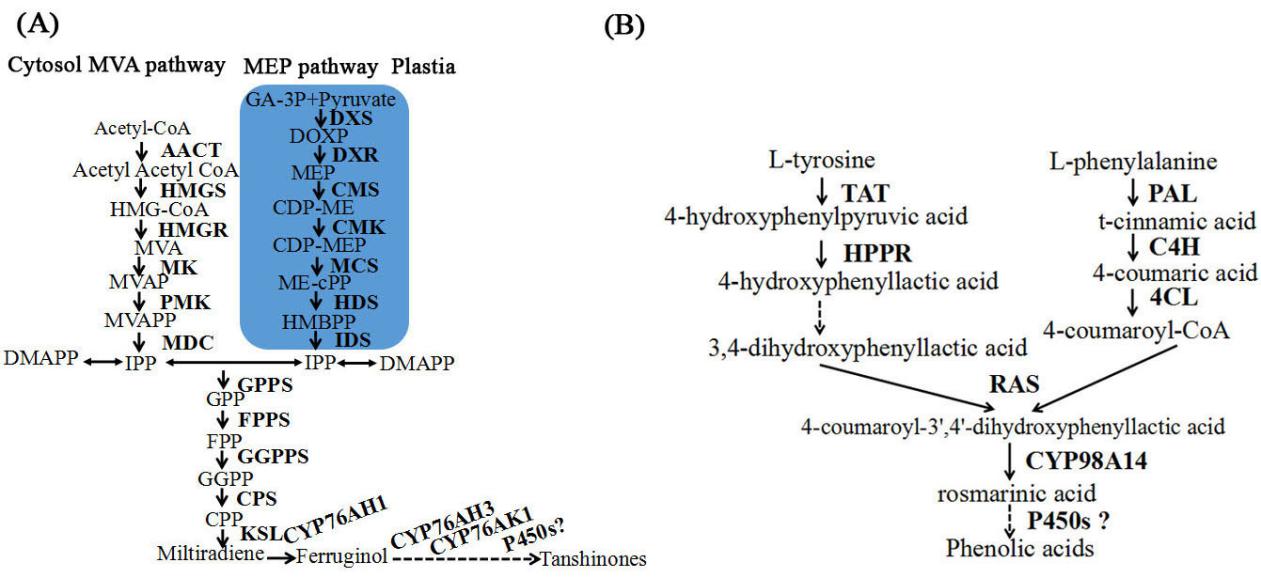


**Supplementary Figure S1.** The biosynthesis pathway of tanshinones and phenolic acids. (A, Tanshinones biosynthesis pathway; B, Phenolic acids biosynthesis pathway)

**
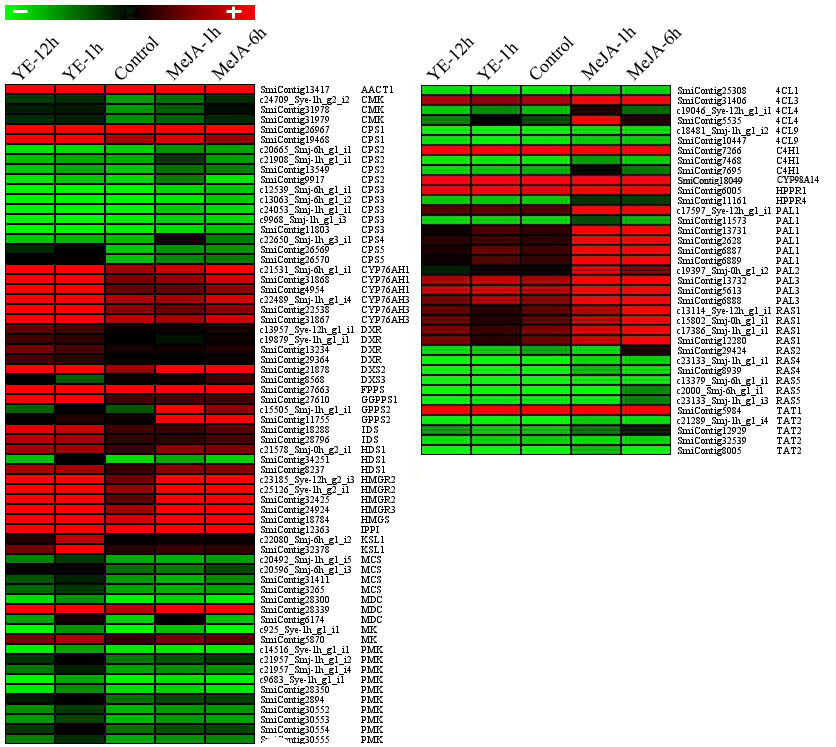
**

**Supplementary Figure S2.** Summary of unigenes involved in the biosynthesis of tanshinones and phenolic acids induced by MeJA and YE.


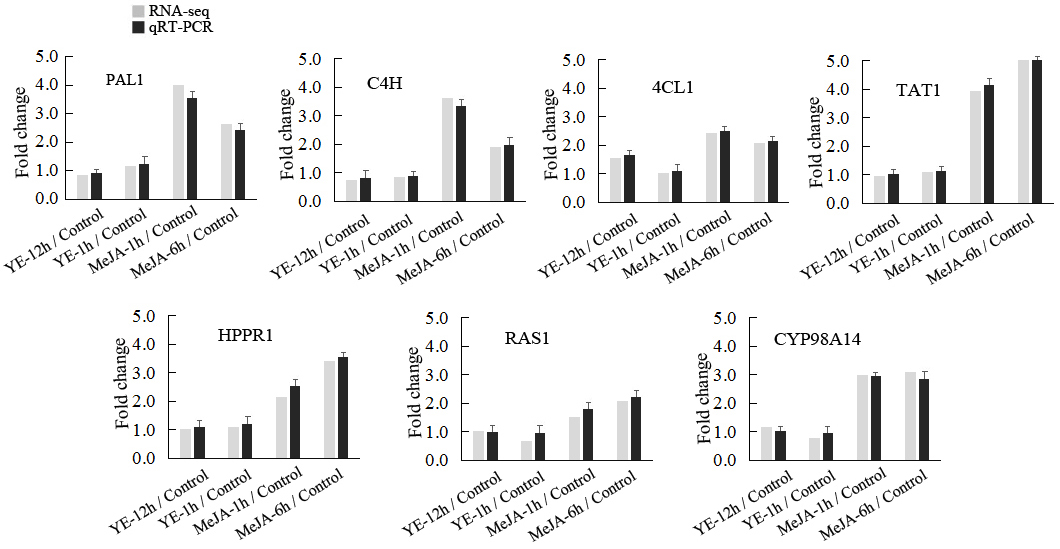


**Supplementary Figure S3.** qRT-PCR validation of differently expressed genes involved in the biosynthesis of phenolic acids in RNA-seq dataset


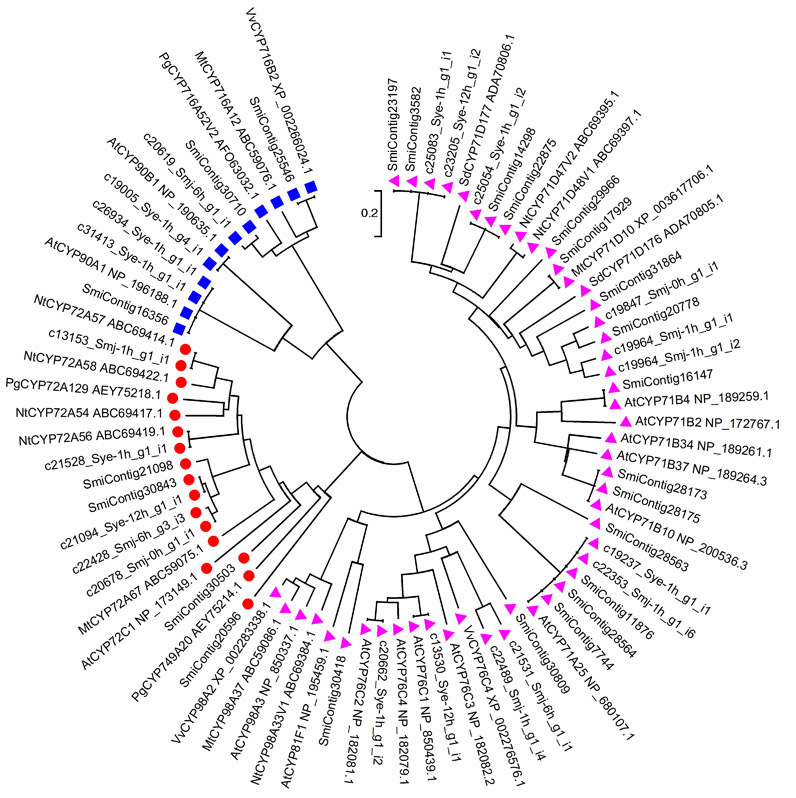


**Supplementary Figure S4.** Phylogenetic analysis of the P450s which co-expressed with genes involved in the biosynthesis of tanshniones. (red solid circle, denotes cytochrome CYP76 clan; blue solid square, denotes cytochrome CYP85 clan; pink solid triangle, denotes cytochrome CYP71 clan)


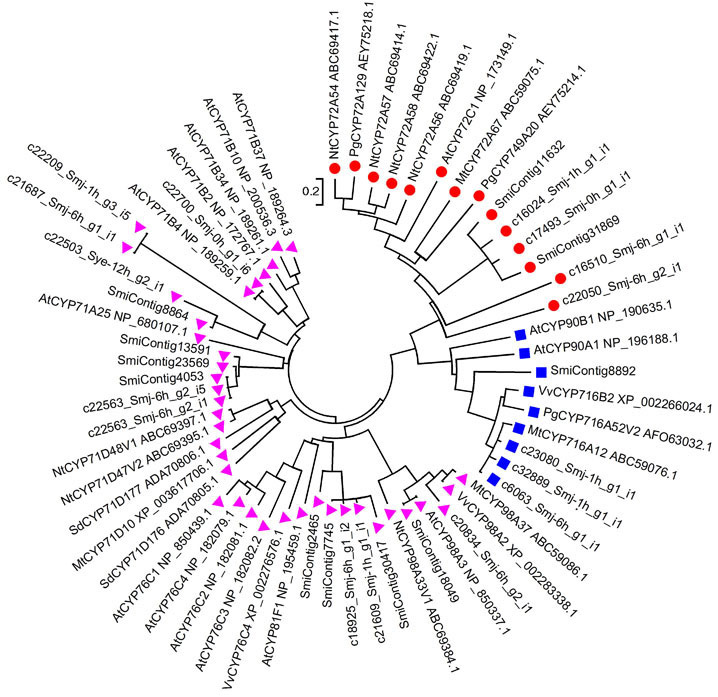


**Supplementary Figure S5.** Phylogenetic analysis of the P450s which co-expressed with genes involved in the biosynthesis of phenolic acids. (red solid circle, denotes cytochrome CYP76 clan; blue solid square, denotes cytochrome CYP85 clan; pink solid triangle, denotes cytochrome CYP71 clan)


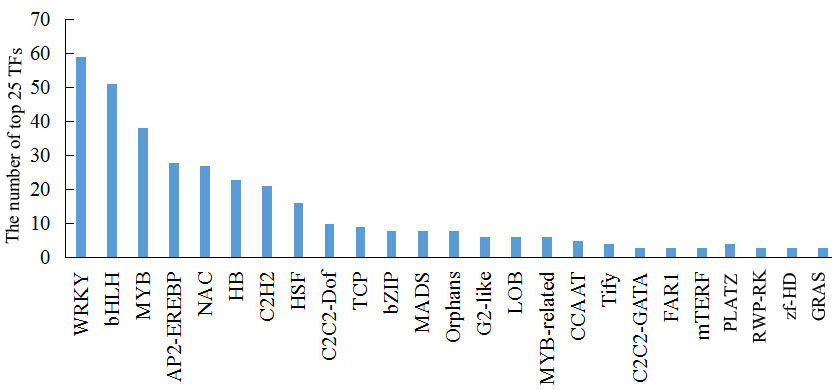


**Supplementary Figure S6.** Summary of top 25 transcription factor families in *S.miltiorrhiza* transcripts
